# Comparison of USG-Guided Supraclavicular Brachial Plexus Block by Single-Point Versus Multiple-Point Injection Technique: A Prospective Randomized Trial

**DOI:** 10.7759/cureus.49018

**Published:** 2023-11-18

**Authors:** Vandana Mangal, Chandrashekhar Meena, Khushboo Meena, Chitra Singh, Tuhin Mistry, Monika Rathore

**Affiliations:** 1 Anesthesiology, Sawai Man Singh Medical College, Jaipur, IND; 2 Anesthesiology and Perioperative Care, Ganga Medical Center & Hospitals Pvt Ltd., Coimbatore, IND; 3 Preventive Medicine, Sawai Man Singh Medical College, Jaipur, IND

**Keywords:** supraclavicular brachial plexus block, ultrasound-guided nerve block, subclavian perivascular brachial plexus block, ropivacaine, brachial plexus block

## Abstract

Background

This study investigated the success rate of ultrasonography (USG)-guided supraclavicular block using a single-point injection technique comparing it with multiple-point injection technique, in terms of nerve sparing, time taken to perform the procedure, time taken to onset of sensory and motor block.

Materials and methods

A total of 204 patients of American Society of Anesthesiologists (ASA) Status I and II, aged 18-60 years, with body mass index (BMI) ≤30 kg/m^2^, posted for upper limb surgeries were given USG-guided supraclavicular brachial plexus block with 15 mL of 0.5% Ropivacaine. The patients were randomly divided into group A (single-point injection) and group B (multiple-point injection), using an out-of-plane technique. Sensory and motor block was assessed for onset and maximum grade achieved, by using pinprick, cold, touch, and movement respectively. The efficacy of the block was tested by assessment in the territories of musculocutaneous, ulnar, radial, median, axillary, and intercostobrachial nerves. Procedural time was calculated from the insertion of the needle till the complete injection of the drug.

Results

Patients in both groups were comparable in terms of demography and ASA status. The success rate for group A was 60.8%, compared to 98% in group B. In group B, the intercostobrachial nerve was most commonly spared (7.84%), followed by ulnar (1.96%), and radial (0.98%). On the other hand, in group A, the most frequently spared nerves were ulnar and intercostobrachial (23.5% each), followed by radial (12.7%), axillary (10.8%), musculocutaneous (7.8%), and median nerves (6.9%). The onset of sensory and motor block was similar in both groups. The procedure time was longer in the multiple-point group.

Conclusion

Our observations suggest that nerve sparing is much lesser in the multiple-point injection technique used for USG-guided supraclavicular block. In a good number of patients using this technique, the intercostobrachial nerve gets blocked.

## Introduction

Supraclavicular brachial plexus block is a popular and widely used regional anesthesia technique for procedures and surgery of the upper extremity. It is an excellent method for attaining optimal operating conditions by producing adequate analgesia, muscular relaxation, and maintaining stable intraoperative hemodynamics. It can be used with ease in emergency and routine surgeries alike. The supraclavicular approach is most frequently used among various approaches of brachial plexus blocks. It is often called the “spinal of the upper limb”. A fairly rapid onset of block is observed following the use of the supraclavicular approach [1].

Ultrasonography (USG) guidance has become the standard of care for nerve blocks. It allows visualization of the underlying structures, needle movement, drug deposition, and spread of the drug. As a result of precise deposition of smaller volumes of local anesthetic, USG, in experienced hands, reduces the incidence of ipsilateral phrenic nerve palsy [2-4].

We hypothesized to assess the difference in success rates of the single-point injection and multiple-point injection techniques of the supraclavicular block. We compared in terms of the sparing of nerves, the time taken to perform the procedure also the differences in the onset of sensory and motor block in the two groups.

## Materials and methods

This prospective, randomized intervention study was conducted in the Department of Anesthesiology and Orthopedics at S.M.S. Medical College, Jaipur, after obtaining due permission from the ethics committee. It was registered with the Clinical Trials Registry of India trial no. CTRI/2022/03/041352. A total of 204 patients of the American Society of Anesthesiologists (ASA status) I & II, aged 18-60 years, with body mass index (BMI) ≤30 kg/m^2^, posted for upper limb surgeries, and satisfying the inclusion criteria were selected. Patients with coagulopathies, any kind of neuropathy, allergy to local anesthetic, pathology at the injection site, and patients not willing to participate in or uncooperative regarding the study were excluded.

Using the opaque sealed envelope method eligible cases were randomly allocated into two study groups: group A (single-point injection technique) and group B (multiple-point injection technique). All patients underwent a thorough pre-anesthetic evaluation and were kept at a nil by-mouth state, according to standard ASA guidelines. Written, informed consent for anesthesia and study was obtained. Baseline vital parameters were recorded in the preoperative room, an IV cannula was secured, and Ringer’s lactate was started. Once patients were received in an operation theater (OT) after being identified, standard vital monitoring was attached (electrocardiogram (ECG), pulse oximeter, and non-invasive blood pressure (BP)).

Blocks were done using the Sonosite Micromax USG machine. The high-frequency linear probe (6-13 Mhz) was prepared using a sterile glove. Patients lay supine with the head turned to the opposite side and the ipsilateral arm adducted. The machine was placed on the same side so that the operator, machine, and patient were in the same line. Painting was done with betadine from the base of the mandible to the ipsilateral areola and from the midline to the axilla. The probe was placed in the supraclavicular fossa. Sterile xylocaine gel was used for contact. Using 2D mode image was optimized to show the first rib, pleura, subclavian artery, and elements of the brachial plexus. 20G 50/100mm stimuplex needle was used out of the plane, the needle path was visualized by tissue movement and hydrodissection having pierced the sheath of the brachial plexus 15mL of 0.5% ropivacaine was injected at one point only in group A. Whereas in group B the same volume 15mL of 0.5% ropivacaine was injected at multiple points in the superior cluster and the corner pocket was separately injected.

The block was not repeated if the intercostobrachial nerve alone escaped. Injection midazolam 0.02mg/kg and fentanyl 1-2 μg/kg were administered to every patient for his/her comfort after assessing the block. Surgery was allowed to proceed as soon as analgesia was achieved in the respective dermatomes. The time to performance of the block was calculated from the time of inserting the needle till the completion of injection. The assessment of block in territories median, ulnar, radial, musculocutaneous, intercostobrachial, and axillary nerves was done at every 2-minute intervals for the first 10 minutes and later every 5 minutes till 30 minutes after completion of the injection of the local anesthetic zero minutes. This was done by using a pinprick for the onset of sensory block [5].

The onset of sensory block was defined as the time from completion of injection of local anesthetic to pinprick achieved till Grade 1. Sensory block was assessed by the following scale - Grade 0: sharp sensation to pinprick, Grade 1: analgesia, dull sensation to pinprick, and Grade 2: anesthesia, no sensation to pin prick.

The motor block was assessed by using the modified Bromage scale [6]. The onset of motor block was taken as the time from the injection of local anesthetic till achievement of motor paralysis equivalent to the modified Bromage Score 1. Any side effects, such as hypotension, bradycardia, nausea, vomiting, and shivering, were noted and treated during the intraoperative and postoperative periods.

Statistical analysis

The primary outcome of our study was identifying the greater success rate of the multiple-point injection technique compared to the single-point injection technique, with reference to USG-guided supraclavicular block. In a previous study conducted by Choudhary et al. [2], using the double-point injection technique increased the rate of success to 96.6% compared to 83.3% using the single-point injection technique. In our study, we used a lower volume of local anesthetic and tried to deposit the drug in different septas around the plexus, using a multiple-point injection technique. We assumed that these two changes in our study design would have a significant effect on the success rate of the block. Based on this, a sample of 92 cases in each group was calculated at 95% confidence and 80% power, to verify the expected difference of 13.4% between the success rates of single-point and multiple-point injection techniques. A 10% loss was considered for the follow-up sample size which was further enhanced to 102. Continuous data was summarized in the form of mean and standard deviations. The difference in the mean of the two groups was analyzed using a student t-test [7]. Continuous data was expressed in the form of proportions and the difference in proportions was analyzed using the chi-square test [8]. The level of significance was maintained at 95% for all statistical analyses.

## Results

**Figure 1:**
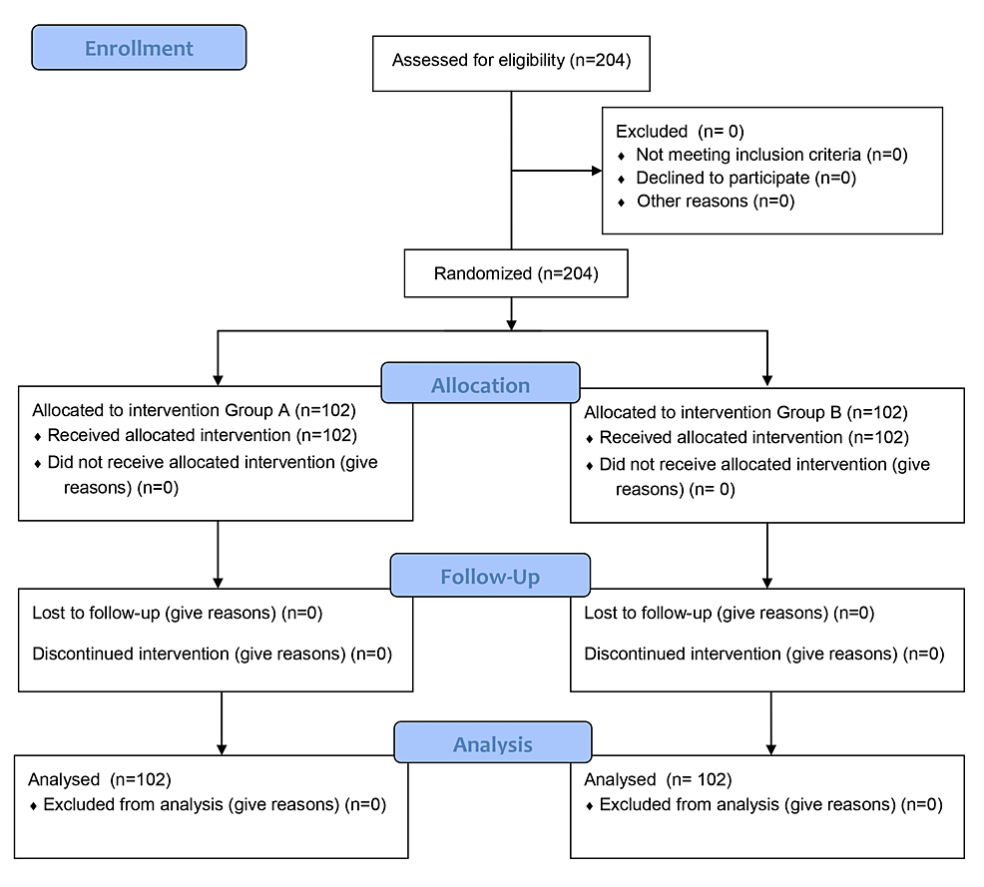
Consort flow diagram

**Figure 2:**
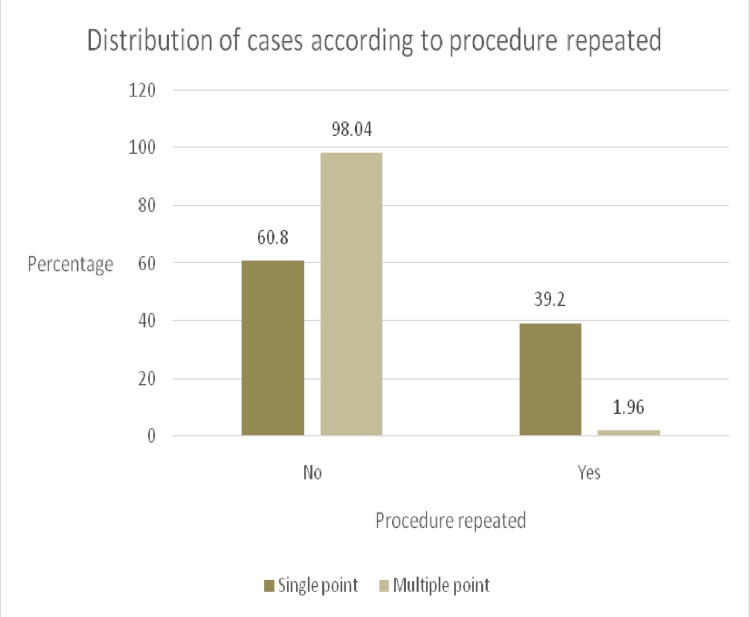
Percentages of procedures repeated in single-point injection and multiple-point injection

**Figure 3:**
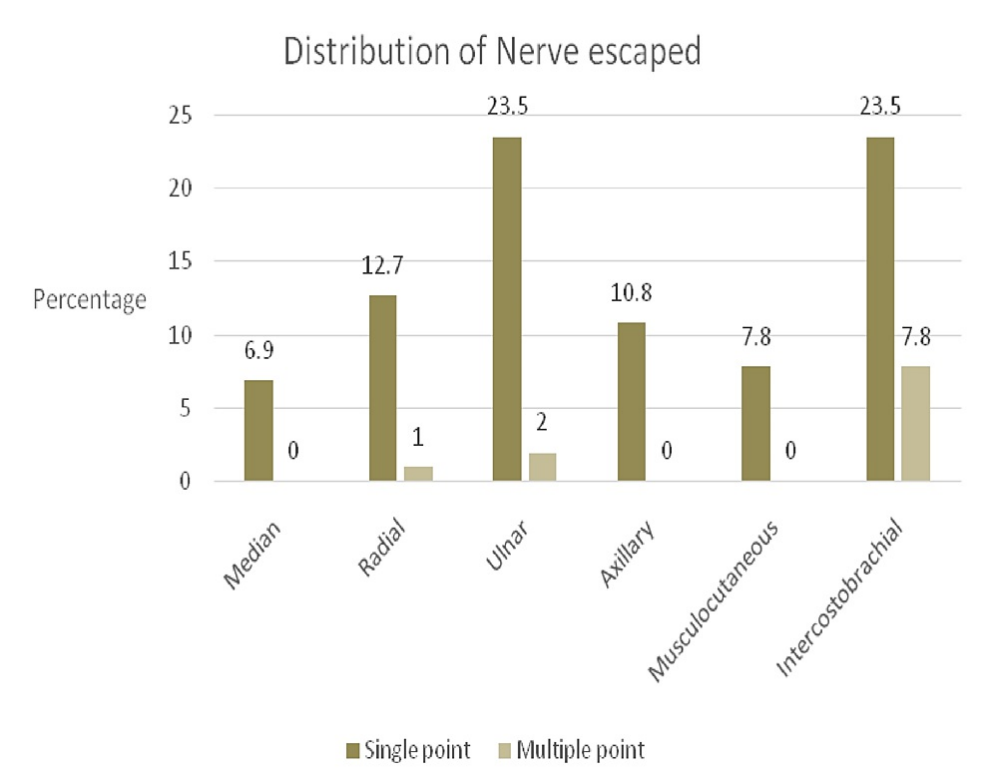
Percentages of nerves missed in single-point injection and multiple-point injection

Patient enrolment in the study is represented in the CONSORT-flow diagram (Figure 1). Patients in both groups were comparable in terms of demographics (Table 1). The success rate in group A was 60.8 % compared to 98% in group B (p-value < 0.05) (Figure 2). In group B, the intercostobrachial nerve was most commonly spared (7.84%), followed by the ulnar (1.96%) and radial (0.98%) nerves. On the other hand, in group A, the most frequently missed nerves were ulnar and intercostobrachial (23.5% each), followed by the radial (12.7%), axillary (10.8%), musculocutaneous (7.8%), and median (6.9%) nerves. There was a statistically significant difference in median, radial, ulnar, axillary, musculocutaneous, and intercostobrachial nerve misses between the two study groups (p-value < 0.05) (Figure 3). The onset of sensory and motor block was similar in both groups (p-value > 0.05); however, the procedure time was longer in the multiple-point group (p-value < 0.001) (Table 2). None of the blocks failed in both groups, and no complications were observed in the groups.

**Table 1:** Mean age, weight, height, and body mass index of cases Continuous variables are presented as means ±SDs; categorical variables are presented as count. BMI indicates body mass index.

Variables	Single point	Multiple point	Test of significance
Age (Years)	34.27±11.96	36.89±13.41	t=1.471, Df=202, p value=0.143
Weight (Kgs)	66.22±8.4	64.94±8.17	t=1.098, Df=202, p value=0.273
Height (Cms)	163.84±7.41	163.32±7.49	t=0.498, Df=202, p value=0.619
BMI	24.66±2.68	24.36±2.75	t=0.795, Df=202, p value=0.428

**Table 2:** Difference in mean of variables P-value compares single-point injection versus multiple-point injection. T-test is used to compare means and chi-square test is used to compare proportion.

Variables	Single-point	Multiple-point	Test of significance
Time to perform procedure (mins)	2.24±0.89	2.78±1.09	t=3.944, Df=202, p value=<0.001
Onset of sensory block (mins)	5.34±2.83	5.02±2.15	t=0.915, Df=202, p value=0.36
Onset of motor block (mins)	11.66±5.65	10.76±3.61	t=1.342, Df=202, p value=0.18

## Discussion

At the end of the day, it is important that we have a successful block in terms of both sensory and motor blockade for anesthesia (solely/as part of GA+RA). Brachial plexus block is a blessing both for the patient and anaesthesiologist for upper limb surgeries. It can be used as a sole anesthetic or in combination with light GA. This study on single vs multiple-point injection of supraclavicular brachial plexus with 15mL of 0.5% Ropivacaine under USG guidance, uses an out-of-plane technique to assess nerve sparing. The time taken to perform the procedure was slightly longer in group B (54sec), which is statistically significant. The most frequently spared nerves were ulnar (23.5%) and intercostobrachial (23.5%) in the single-point injection group, despite the deposition of the local anesthetic well within the brachial plexus sheath. On the other hand, in the multiple-point technique, the frequency of nerve-sparing was very low: ulnar (1.96%), radial (0.98%), intercostobrachial (7.8%). In the multiple-point technique, we ensured that all nerve elements were surrounded by the local anesthetic, thus ensuring the deposition of the drug beyond various septas. The single-point technique was found to be technically simple and faster, producing comparable motor and sensory onset of anesthesia. However, our observations are similar to Choudhary et al. [2] in that despite USG guidance, the success rate was not 100% possible due to some septal barriers. Multiple-point injection of supraclavicular brachial plexus through USG guidance ensured a success rate with minimal sparing. Another interesting observation was that the majority of the patients reported anesthesia in the territory of the intercostobrachial nerve too. In contrast to our study, Vallapureddy et al. [9] found that sensory and motor blockades of the musculocutaneous, median, radial, and ulnar nerve distribution were similar in both groups. Choi et al. [10] and Arab et al. [11] found that the rate of blockage of nerves was lower in the single-point injection group, which is similar to our study. Choi et al. [10], Choudhary et al. [2], Vallapureddy et al. [9], Pratap et al. [12], Tran et al. [13], and Arab et al. [11] also observed that the single-point injection technique takes lesser procedure time. Choudhary et al. [2], Jung et al. [10], Murali et al. [14], Tran et al. [13], Pratap et al. [12], Sherin et al. [15], Arab et al. [11], and Vallapureddy et al. [9] all found delayed sensory and motor onset in the single-point injection group, compared to double- or triple-injection groups.

None of the patients clinically reported dyspnea, fall in blood oxygen (SpO_2_) levels, hypotension, bradycardia, vomiting, vessel puncture, pneumothorax, or signs of respiratory distress. In our study, we observed that the multiple-point injection technique of the supraclavicular block had a better success rate as compared to the single-point injection technique. If the local anesthetic was carefully deposited within the brachial plexus sheath under USG guidance, there were fewer chances of block failure. We have not used PNS, using dual guidance could have offered a 100% success rate. However, these could be a limitation of our study.

## Conclusions

Nerve-sparing was seen more frequently in single-point injection, but the onset of sensory and motor block was similar to the multiple-point injection group. In the single-point injection group, the frequently missed nerves were ulnar, axillary, radial, and musculocutaneous, whereas in the multiple-point injection group, ulnar and radial nerves were missed. Interestingly, we observed that the intercostobrachial nerve was blocked in 92.2% cases in the multiple-point group and in 76.5% cases in the single-point group.
